# Amycenone reduces excess body weight and attenuates hyperlipidaemia by inhibiting lipogenesis and promoting lipolysis and fatty acid β-oxidation in KK-*A^y^* obese diabetic mice

**DOI:** 10.1017/jns.2022.43

**Published:** 2022-07-11

**Authors:** Maya Kudo, Misa Hayashi, Boju Sun, Lili Wu, Tonghua Liu, Ming Gao

**Affiliations:** 1 School of Pharmaceutical Science, Mukogawa Women's University, 11-68 Koshien Kyuban-cho, Nishinomiya, Hyogo 663-8179, Japan; 2 Second Clinical Medical College, Beijing University of Chinese Medicine, No. 11 North Third Ring East Road, Beijing 100029, China; 3 Key Laboratory of Health Cultivation of the Ministry of Education, Beijing University of Chinese Medicine, No. 11 North Third Ring East Road, Beijing 100029, China; 4 College of Traditional Chinese Medicine, Beijing University of Chinese Medicine, No. 11 North Third Ring East Road, Beijing 100029, China; 5 Institute for Bioscience, Mukogawa Women's University, 11-68 Koshien Kyuban-cho, Nishinomiya, Hyogo 663-8179, Japan

**Keywords:** Amycenone, Hepatic fat accumulation, Lipid metabolism pathway, Obese/diabetes KK-*A^y^*/TaJcl mice

## Abstract

Excess body weight and hyperlipidaemia cause severe health problems and have social implications. Amycenone is an active substance extracted from Yamabushitake mushrooms with no reports of its activity against excess body weight and hyperlipidaemia. This research clarifies the effects and mechanisms of action of amycenone on the inhibition of body weight excess and hyperlipidaemia attenuation using KK-*A^y^* mice. Amycenone or water was administered to 8-week-old male KK-*A^y^* mice by gavage for 8 weeks. Their body weight and food intake were recorded during the experiment. At the end of the experimental period, the mice were dissected, and blood samples, lipid metabolism-related organs and tissues were collected and stored for further analysis. Amycenone treatment suppressed body weight gain and improved serum levels of fasting blood glucose and non-esterified fatty acids. Additionally, serum and hepatic cholesterol and triacylglycerol levels were reduced after this treatment, whereas the phosphorylation levels of AMPK, PKA and HSL increased and the expression level of FAS decreased. The protein level of C/EBPβ and gene expression level of *Cpt1* were higher in the perirenal adipose tissue of amycenone-treated KK-*A^y^* mice. Furthermore, amycenone phosphorylated AMPK, PKA and ACC, and PPARγ expression was lower in the mesenteric adipose tissue. The phosphorylation levels of AMPK, LKB1, PKA and ACC were also induced, and FAS expression level was reduced in the liver of the amycenone-treated group. Amycenone could reduce excess body weight and attenuate hyperlipidaemia in KK-*A^y^* mice by inhibiting lipogenesis and promoting lipolysis through lipid metabolism pathway stimulation and fatty acid β-oxidation acceleration.

## Introduction

The progression of obesity is a critical health issue worldwide. According to a recent report, approximately half of the adult population is considered overweight or obese in over eleven countries of the Organization for Economic Cooperation and Development (OECD)^(1)^. Obesity refers to the excessive accumulation of fat, causing hyperlipidaemia, hypertension, type 2 diabetes (T2D), cardiovascular disease and non-alcoholic fatty liver disease (NAFLD), and is involved in metabolic syndrome development^(2–5)^. Obesity is caused by excessive calorie intake and low energy expenditure^(6)^, resulting in induced lipid accumulation in various organs and tissues, such as liver and adipose tissue^(7)^. Therefore, when aiming to develop novel treatments against obesity, it is essential to select two desirable outcomes: body weight reduction and hyperlipidaemia control^(8)^.

Several factors associated with lipid metabolism signalling pathways have been previously reported. AMP-activated protein kinase (AMPK) is a major factor in cell regulation.

AMPK is a significant regulator of cell metabolic energy homeostasis^(9–12)^. AMPK activation is involved in regulating energy and nutrient metabolism, including the synthesis of fatty acids, cholesterol and glucose, as well as hepatic gluconeogenesis and translation^(9)^. Collectively, AMPK is considered a crucial target factor in preventing obesity, diabetes and inflammation^(10–13)^. AMPK phosphorylation is primarily regulated by two enzymes, which are calcium/calmodulin-dependent protein kinase kinase (CaMKK) and liver kinase B1 (LKB1)^(14,15)^. AMPK also regulates the transcription of peroxisome proliferation-activated receptor γ (PPARγ), CCAAT/enhancer-binding protein (C/EBP) family members and sterol regulatory element-binding protein 1 C (SREBP1C). These enzymes and transcription factors regulate acetyl-CoA carboxylase (ACC) expression, which can be inactivated by phosphorylation, fatty acid synthase (FAS) and hormone-sensitive lipase (HSL), involved in lipogenesis or lipolysis^(16,17)^.

Previous studies have reported that AMPK activation promotes β-oxidation of white adipose tissue (WAT) and inhibits lipogenesis, and it is involved in obesity and insulin resistance^(18,19)^. It has already been reported that the promotion of energy expenditure by β-oxidation of WAT plays a significant role in improving obesity and metabolism in rodents and humans^(18,20,21)^. Additionally, obese subjects have a higher amount of accumulated triacylglycerol (TG) and abnormal lipid metabolism compared to non-obese subjects^(22)^.

Obese/diabetes KK-*A^y^*/TaJcl (KK-*A^y^*) mice were established in 1969^(23)^. KK-*A^y^* mice were created by introducing the yellow obesity gene (*A^y^* allele) into KK mice. KK-*A^y^* mice developed more severe diabetes than KK mice and have been widely selected as model animals for research on T2D and obesity. It has been published that L-citrulline, a free amino acid, is detected mainly in watermelon ameliorated obesity^(24,25)^ using the KK-*A^y^* mice model^(26)^. Therefore, the KK-*A^y^* mice are suitable models for studies on obesity and hyperlipidaemia^(27–32)^.

Various natural products have been reported to improve obesity and metabolism through AMPK activation^(19,33–35)^. Yamabushitake mushroom (*Hericium erinaceus*) is a popular edible mushroom in the temperate region of the northern hemisphere, including Japan and China, and it is utilised as a traditional Chinese medical mushroom for over 400 years^(36)^. Yamabushitake mushroom composition includes β-glucan, dietary fibres, vitamins, amino acids and minerals. Amycenone is an extract from Yamabushitake, containing 6 % amyloban and 0⋅5 % hericenone. Amyloban and hericenone are active ingredients contained in Yamabushitake mushroom, and amycenone is standardised to a certain amount. Amyloban is a bioactive fat-soluble component that prevents cell death due to abnormal proteins that are said to be the cause of Alzheimer-type dementia. Herisenone is an active compound involved in the youth and health of nerve cells and has been confirmed to increase nerve growth factor^(37)^. Other additives in amycenone are excipients, stabilisers, binders, preservatives, disintegrants and sweeteners. It is recognised that the addition of these additives do not affect the active substances and can be used in pharmaceuticals and health foods. Amycenone is believed to improve dementia, depression and insomnia, including sleep apnoea and suppress ‘brain aging’^(38)^. However, only a few studies report its effects on obesity and lipid metabolism.

The present study reveals the effects of amycenone on excess body weight and hyperlipidaemia and its mechanism using KK-*A^y^* obese diabetic mice.

## Methods

### Amycenone preparation

Sun Medica Co. Ltd. (Tokyo, Japan) provided amycenone. Amycenone dose for administration to KK-*A^y^* mice was determined by comparison with a human weighting 50 kg, taking a daily dose of 1.9 g amycenone daily and used twenty times in KK-*A^y^* mice as human daily intake^(39)^. Amycenone was dispersed in tap water. The experiments were terminated once amycenone could induce bodyweight reduction.

### Experimental animals and supplementation with amycenone

Seventeen six-week-old male obese/diabetic KK-*A^y^* mice were purchased from CLEA Japan, Inc., (Tokyo, Japan) and housed between 22 and 24°C under light/dark cycle for 12/12 h. CE-2 (standard diet; CLEA Japan Inc., (Tokyo, Japan)) was administered to the mice for 2 weeks to stabilise their metabolic state. After, mice were randomly separated into two groups: the control group (standard diet; normal water by gavage, *n* 9) and the amycenone group (standard diet; 0.76 g/kg body weight amycenone/day by gavage, *n* 8). Amycenone or normal water was orally administered to KK-*A^y^* mice for 8 weeks. Their body weight, food intake and water intake were measured once a week. Blood was collected once a month, and an oral glucose tolerance test (OGTT) was performed 2 months after the initiation of the administration.

After all amycenone administration are completed, all mice were dissected after fasting for 12–18 hours. The dissection is performed on a different schedule from blood collection and OGTT so that it will not be affected by the fasting time. Mice were anaesthetised with isoflurane (FUJIFILM Wako Pure Chemical Corporation, Osaka, Japan), and utmost efforts, including sacrifices by highly skilled researchers, were made to minimise pain. Blood was collected from the heart, and plasma and serum were picked up by centrifugation and cryopreserved at −20°C for future research. Various organs and tissues, including the liver, heart, kidneys, perirenal adipose tissue (PAT), epididymal adipose tissue (EAT), mesenteric adipose tissue (MAT), subcutaneous adipose tissue (SAT) and brain, were removed, and their weights were measured. The removed organs and tissues were rapidly frozen in liquid nitrogen and stored at −80°C for use in western blotting and real-time polymerase chain reaction (PCR) analysis.

The methods of all animal experiments followed the guidelines for animal care and use in the field of physiology established by the Physiological Society of Japan and the ARRIVE guidelines. Additionally, the animal experiments were examined by the Experimental Animal Ethics Committee of Mukogawa Women's University and conducted after approval (permit number: P-06-2019-01-A).

### Biochemical analysis of serum and liver tissue extracts

Fasting blood glucose (FBG) level was determined using a self-testing glutest sensor (Sanwa kagaku kenkyusho Co. Ltd., Aichi, Japan). TG and cholesterol (TC) in serum and liver, serum non-esterified fatty acid (NEFA), aspartate aminotransferase (AST) and alanine aminotransferase (ALT) were measured using a commercially available assay kit (FUJIFILM Wako Pure Chemical Corporation, Osaka, Japan). Liver TG and TC levels were expressed as mg/g (liver weight) by dividing the calculated value by the liver weight^(39)^.

### Oral glucose tolerance test

After a 12–18 hours fasting, OGTT was performed shortly before the end of the 8-week experimental period. FBG was measured using blood from the tail vein every 0, 30, 60, 90 and 120 min after glucose loading (gavage with 1 g glucose/kg body weight). The area under the curve (AUC) was calculated from the hourly glucose level^(40)^.

### Protein isolation experiments

EAT, PAT, MAT, SAT and liver tissue were incubated in homogenisation buffer (50 mM Tris-HCl (pH 7⋅4), 100 mM NaCl, 1 % NP-40, 0⋅25 % Na deoxycholate, 0⋅1 % SDS, 1 mM EDTA, 50 mM NaF, 2 mM Na_3_VO_4_, 30 mM Na pyrophosphate and 2 mM PMSF) for protein isolation. After incubation on ice for 30 min, the extract was centrifuged at 12 000 rpm for 10 min to isolate the supernatant. Tissues were heat-treated at 100°C for 2⋅5 min with 2X SDS sample buffer (0⋅5 M Tris-HCl (pH 6⋅8), glycerol, SDS, 1 % bromophenol blue and 2-mercapto ethanol) and used as a sample for protein analysis^(26,41)^.

### Primary and secondary antibodies

Primary and secondary antibodies against AMPK, phosphor-AMPK, phosphor-HSL, FAS, CaMKK, phosphor-CaMKK, LKB1, phosphor-LKB1, protein kinase A (PKA), phosphor-PKA, Sirtuin 1 (Sirt1), PPARγ, C/EBPα, C/EBPβ, anti-rabbit IgG and anti-mouse IgG were purchased from Cell Signalling Technology (Commonwealth of Massachusetts, USA). Phosphor-C/EBPβ was purchased from Abcum (Cambridge, UK). HSL and anti-mouse β-actin were purchased from Sigma (State of Missouri, USA).

### Western blotting

Proteins (100 μg/lane) were electrophoresed at 100 V for 2 hours using a 12⋅5 % SDS-PAGE gel for separation. Next, the proteins were transferred to a polyvinylidene difluoride membrane (PVDF) (Amersham Life Science Inc., Commonwealth of Massachusetts, USA) at 100 mA for 2 hours. The membrane was incubated with Blocking One or Blocking One-P solution (Nacalai Tesque, Kyoto, Japan) for 30 min, and then the primary antibody diluted 1:1000-500 with Can Get Signal Solution 1 (Toyobo, Osaka, Japan) was subjected to a reaction at 4°C overnight. The next day, the membrane was washed with TBST containing 1 M Tris-HCl (pH 7⋅5), NaCl and 20 % Tween 20, and then the anti-rabbit or mouse horseradish peroxidase-conjugated IgG diluted 1:10 000-2000 with Can Get Signal Solution 2 (Toyobo, Osaka, Japan) was subjected to an antibody reaction at room temperature for 1 hour.

Protein band detection was performed using Chemi-Lumi One Super (Nacalai Tesque, Kyoto, Japan) and Ez-Capture ST (ATTO Corporation, Tokyo, Japan). β-actin was used as a loading control. Protein band intensity analysis was conducted using ImageJ public domain software (National Institutes of Health, State of Maryland, USA)^(26,41)^.

### RNA extraction and real-time PCR

Total-RNA was extracted from PAT, MAT and liver using Sepasol (R)-RNA I Super G (Nacalai Tesque, Kyoto, Japan) and absorbances 260 and 280 using a branch photometer (Hitachi, Tokyo, Japan). The extracted RNA is reverse transcribed into cDNA using the Rever Tra Ace qPCR RT Master Mix with g DNA remover (Toyobo, Osaka, Japan), and then real-time PCR was performed using THUNDERBIRD Next SYBR qPCR Mix (Toyobo, Osaka, Japan), and the relative expression level of the target gene was examined. Specific primers were purchased and used, which were synthesised by Thermo Fisher Scientific (Commonwealth of Massachusetts, USA) (Table 1). The RNA amplification reaction was conducted using a thermal cycler dice (Takara Bio Inc., Shiga, Japan) under the conditions of one cycle for 30 s at 95°C, 40 cycles for 5 s at 95°C and 30 s at 60°C. Expression levels of mRNA levels were analysed using the 2^–ΔΔCT^ method. Relative expression levels of mRNA were determined by calculating the ratio of each transcript to transcript using glyceraldehyde-3-phosphate dehydrogenase (GAPDH) as the housekeeping gene^(40,41)^.

**Table 1.** Specific primer sequences of lipid metabolism-related genes

Genes	Forward	Reverse
*Gapdh*	AGAACATCATCCCTGCATCCA	CCGTTCAGCTCTGGGATGAC
*Atgl*	GGTGCCAACATTATTGAGGTG	AAACACGAGTCAGGGAGATGC
*Aco*	CCCAAGACCCAAGAGTTCATTC	CACGGATAGGGACAACAAAGG
*Mcad*	TGTGCCTACTGCGTGACAGA	TTCATCACCCTTCTTCTCTGCTT
*ap2*	CAAAATGTGTGATGCCTTTGTC	CTCTTCCTTTGGCTCATGCC
*Ppara*	TGGAGTCCACGCATGTGAAG	TGTTCCGGTTCTTTTTCTGAATCT
*Cpt1*	CGGTTCAAGAATGGCATCATC	ATCACACCCACCACCACGATA
*Adipor1*	TGAGGTACCAGCCAGATGTC	CGTGTCCGCTTCTCTGTTAC
*Adipor2*	TCCATGGAGTCTCAAACCTG	GGAGAGTATCACAGCGCATC
*Tnf-a*	AAATGGGCTCCCTCTCATCAGTTC	TCTGCTTGGTGGTTTGCTACGAC
*Il-6*	CAGTGTCATGGTTCCTTTGC	CACCGAGGAACTACCTGAT
*Il-1b*	CACCTCTCAAGCAGAGCACAG	GGGTTCCATGGTGAAGTCAAC

### Specific primer sequences

The following purchased specific primers were used for real-time PCR: *Gapdh*, adipose triacylglycerol lipase (*Atgl*), acyl-CoA oxidase (*Aco*), middle-chain acyl-CoA dehydrogenase (*Mcad*), adipocyte protein 2 (*ap2*), *Ppara*, carnitine palmitoyltransferase 1 (*Cpt1*), adiponectin receptor 1 (*Adipor1*), adiponectin receptor 2 (*Adipor2*), tumour necrosis factor α (*Tnf-a*), interleukin 6 (*Il-6*) and interleukin 1β (*Il-1b*). They were all purchased from Thermo Fisher Scientific (Commonwealth of Massachusetts, USA) (Table 1).

### Statistical analysis

Experimental data were expressed as mean ± standard error, and statistical analysis was performed using Student's t test for a significant difference between the two groups, and a P-value was less than 0⋅05 and judged to be statistically significant. All animals were included in the analysis.

## Results

### Amycenone reduced body weight, body weight gain and liver weight in KK-*A^y^* mice

**Fig. 1.**
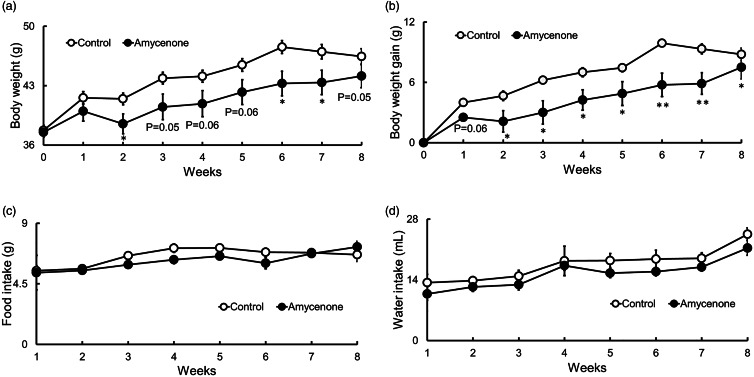
Body weight, body weight gain and food and water intakes in KK-*A^y^* mice with normal water or under amycenone treatment (0.76 g/kg body weight/day) for 8 weeks. Amycenone reduced body weight and body weight gain in KK-*A^y^* mice. (a) Body weight, (b) body weight gain, (c) food intake and (d) water intake. White circles represent the control group and black circles represent the amycenone group. The data value is given as means ± sem (*n* 9, 8, respectively). *P < 0⋅05, **P < 0⋅01 *v*. control group.

First, the effects of amycenone on body weight, body weight gain, food intake and water intake in KK-*A^y^* mice were investigated. When mice were treated with amycenone or water for 8 weeks, both body weight and body weight gain tended to be lower in the amycenone group (Fig. 1(a) and (b)). Additionally, food and water intake did not significantly change between both groups (Fig. 1(c) and (d)). The liver weight significantly decreased in mice belonging to the amycenone group, with no other organ or tissue weights varying between the two study groups (Table 2).

**Table 2.** Organ and tissue weights in KK-*A^y^* mice with normal water or under amycenone treatment (0.76 g/kg body weight/day) for 8 weeks

Organ and tissue	Control (mg/g (body weight))	Amycenone (mg/g (body weight))
Liver	50⋅8 ± 0⋅63	46⋅0 ± 1⋅58*
Kidneys	11⋅8 ± 0⋅37	13⋅3 ± 0⋅20
Heart	4⋅0 ± 0⋅21	4⋅3 ± 0⋅16
EAT	30⋅1 ± 0⋅92	30⋅8 ± 2⋅36
Brain	7⋅0 ± 0⋅14	7⋅4 ± 0⋅55

Amycenone decreased liver weight in KK-*A^y^* mice. The table shows the weights of the liver, kidneys, heart, EAT and brain. The data value is given as means ± sem (*n* 9, 8, respectively).

* *P* < 0⋅05 *v*. control group.

### Amycenone reduced serum levels of FBG, TG, TC and NEFA and hepatic levels of TG and TC in KK-*A^y^* mice

**Fig. 2.**
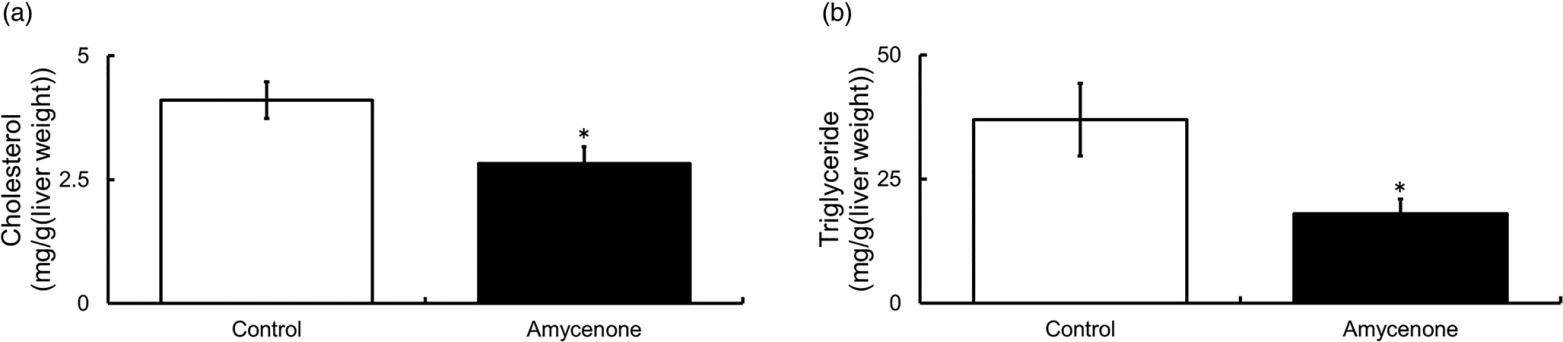
Hepatic levels of TC and TG in KK-*A^y^* mice with normal water or under amycenone treatment (0.76 g/kg body weight/day) for 8 weeks. Amycenone decreased hepatic levels of TC and TG in KK-*A^y^* mice. (a) TC and (b) TG. White bars represent the control group and black bars represent the amycenone group. The data value is given as means ± sem (*n* 9, 8, respectively). *P < 0⋅05 *v*. control group.

Various serum and hepatic metabolic parameters in KK-*A^y^* mice were evaluated. Serum FBG level was significantly lower at 4 weeks in the amycenone group, whereas serum TC and TG levels were significantly lower at 8 weeks after the initiation of the amycenone treatment. Furthermore, the serum level of NEFA decreased significantly at 4 weeks and tended to decrease at 8 weeks (*P* = 0⋅08) (Table 3). Moreover, hepatic TC and TG levels significantly decreased in the amycenone group (Fig. 2(a) and (b)); however, serum AST and ALT, markers of hepatic function, did not change throughout the treatment (Table 3).

**Table 3.** Metabolic parameters in serum and liver of KK-*A^y^* mice with normal water or under amycenone treatment (0.76 g/kg body weight/day) for 4 and 8 weeks

	Weeks	Control	Amycenone
FBG (mg/dl)	4	179⋅4 ± 10⋅7	146⋅0 ± 8⋅89*
	8	168⋅3 ± 8⋅51	163⋅9 ± 14⋅7
TC (mg/dl)	4	87⋅4 ± 5⋅18	82⋅4 ± 9⋅12
	8	91⋅9 ± 4⋅07	74⋅0 ± 5⋅39*
TG (mg/dl)	4	315⋅3 ± 29⋅7	229⋅5 ± 29⋅0
	8	119⋅0 ± 4⋅80	82⋅6 ± 11.0*
NEFA (mEq/l)	4	1⋅50 ± 0⋅09	1⋅13 ± 0⋅12*
	8	0⋅99 ± 0⋅06	0⋅82 ± 0⋅07
AST (IU/l)	4	59⋅0 ± 3⋅35	62⋅5 ± 5⋅52
	8	79⋅9 ± 3⋅87	96⋅4 ± 16⋅2
ALT (IU/l)	4	7⋅69 ± 0⋅92	6⋅48 ± 0⋅89
	8	11⋅6 ± 0⋅65	11⋅1 ± 1⋅28

Amycenone decreased the serum levels of FGB, TG, TC and NEFA in KK-*A^y^* mice. The table shows the serum levels of FGB, TG, TC, NEFA, AST and ALT. The data value is given as means ± sem (*n* 9, 8, respectively).

* *P* < 0⋅05 *v*. control group.

### Amycenone improved glucose tolerance in KK-*A^y^* mice

**Fig. 3.**
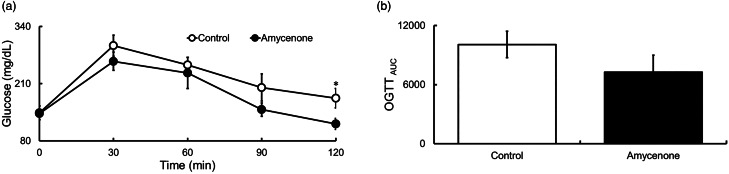
Blood glucose levels and AUC in KK-*A^y^* mice with normal water or under amycenone treatment (0.76 g/kg body weight/day) during 1⋅0 g/kg body weight OGTT in 8 weeks. Amycenone time-dependently improved glucose tolerance. (a) Blood glucose and (b) AUC. White circles and bars represent the control group and black circles and bars represent the amycenone group. The data value is given as means ± sem (*n* 9, 8, respectively). *P < 0⋅05 control group.

OGTT revealed that amycenone can time-dependently inhibit blood glucose elevation, specifically 120 min after glucose tolerance (Fig. 3(a) and (b)).

### Amycenone affected the phosphorylation of AMPK in EAT, SAT, PAT and MAT in KK-*A^y^* mice

Western blotting was performed to examine the phosphorylation level of AMPK, a significant factor in lipid metabolism. To clarify the mechanism of inhibition of body weight gain with amycenone treatment, four types of adipose tissues, including EAT, SAT, PAT and MAT, were used. Amycenone did not affect AMPK phosphorylation of EAT (Supplementary Fig. S1(a) and (g)) and SAT (Supplementary Fig. S1(b) and (h)); however, AMPK phosphorylation levels of PAT (Supplementary Fig. S1(c)–(e) and (i)–(k)) and MAT (Supplementary Fig. S1(f) and (l)) significantly increased. Thus, PAT and MAT were selected for examining the mechanism of inhibition of body weight gain with amycenone treatment in the following experiments.

### Amycenone affected the phosphorylation and expression levels of AMPK-mediated signalling-related proteins in PAT of KK-*A^y^* mice

Since the activation level of AMPK was significantly higher in PAT in mice in the amycenone group, the phosphorylation and expression levels of AMPK-mediated signalling-related proteins, FAS, ACC and HSL, which are downstream regulators of AMPK, were measured. There was no change in the FAS level (Supplementary Fig. S2(a) and (h)), whereas ACC and HSL phosphorylation were significantly higher in the amycenone group (Supplementary Fig. S2(b), (c), (i) and (j)). Next, CaMKK, LKB1 and PKA, which are upstream regulators of AMPK, were examined. It was discovered that the phosphorylation of CaMKK and LKB1 did not change with amycenone treatment (Supplementary Fig. S2(d), (e), (k) and (l)), while PKA was significantly activated in this group (Supplementary Fig. S2(f) and (m)). Finally, the phosphorylation level of C/EBPβ, a lipid metabolism-related transcription factor, were upregulated in mice under amycenone treatment (Supplementary Fig. S2(g) and (n)).

### Amycenone affected the expression levels of lipid metabolism-related genes in PAT of KK-*A^y^* mice

The effect of amycenone on the expression level of genes associated with lipid metabolism in PAT of KK-*A^y^* mice was examined. Real-time PCR was performed to analyse the mRNA levels of *Atgl*, *Mcad*, *ap2*, *Cpt1*, *Adipor1* and *Adipor2*. Amycenone treatment did not affect the mRNA levels of *Mcad*, *ap2*, *Adipor1* and *Adipor2*, while the mRNA level of *Cpt1* was significantly higher, indicating that amycenone promoted β-oxidation and thermogenesis by upregulating of *Cpt1* level (Supplementary Table S1).

### Amycenone affected the phosphorylation and expression levels of AMPK-mediated signalling-related proteins in MAT of KK-*A^y^* mice

The phosphorylation and expression levels of AMPK-mediated signalling-related proteins were measured because the phosphorylation level of AMPK was significantly higher in the MAT of the amycenone group. HSL (Supplementary Fig. S3(a) and (j)) and FAS levels (data not shown) did not change between the two groups. Conversely, ACC phosphorylation was significantly higher in the amycenone group (Supplementary Fig. S3(b) and (k)). Furthermore, CaMKK, LKB1, Sirt1 and PKA, which have been reported as upstream regulators for AMPK, were analysed. CaMKK phosphorylation was discovered to be higher in amycenone-treated mice (Supplementary Fig. S3(c) and (l)); however, LKB1 and Sirt1 were unaltered (Supplementary Fig. S3(d), (e), (m) and (n)). On the other hand, PKA phosphorylation was significantly higher with amycenone treatment (Supplementary Fig. S3(f) and (o)). Finally, the expression levels of PPARγ, C/EBPα and the phosphorylation level of C/EBPβ, which are transcription factors associated with lipid metabolism, were investigated. While C/EBPα and C/EBPβ levels remained unchanged in amycenone-treated mice (Supplementary Fig. S3(h), (i), (q) and (r)), the PPARγ level was reduced (Supplementary Fig. S3(g) and (p)).

### Amycenone affected the phosphorylation and expression levels of lipid metabolism-related proteins in the liver of KK-*A^y^* mice

**Fig. 4.**
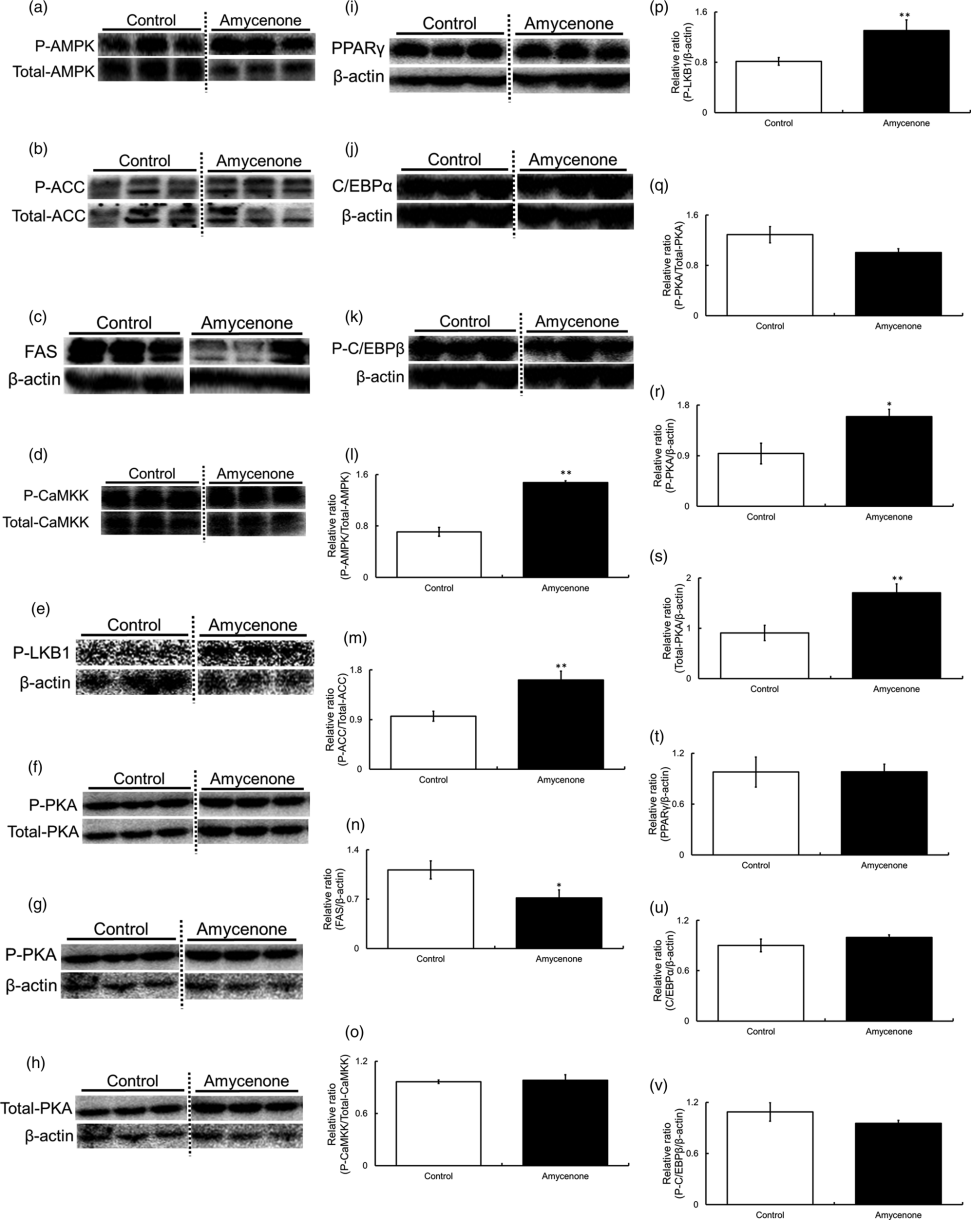
The phosphorylation and expression levels of AMPK, ACC, FAS, CaMKK, LKB1, PKA, PPARγ, C/EBPα and C/EBPβ in the liver of KK-*A^y^* mice with normal water or under amycenone treatment (0.76 g/kg body weight/day) for 8 weeks. Amycenone increased the phosphorylation levels of AMPK, ACC, LKB1 and PKA and decreased the expression level of FAS in the liver of KK-*A^y^* mice. (a, l) AMPK, (b, m) ACC, (c, n) FAS, (d, o) CaMKK, (e, p) LKB1, (f–h, q–s) PKA, (i, t) PPARγ, (j, u) C/EBPα and (k, v) C/EBPβ. White bars represent the control group and black bars represent the amycenone group. The data value is given as means ± sem (*n* 9, 8, respectively). *P < 0⋅05, **P < 0⋅01 *v*. control group.

As amycenone affected hepatic fat accumulation in KK-*A^y^* mice, western blotting was performed to examine the expression level of proteins involved in lipid metabolism in the liver. AMPK phosphorylation was significantly higher in amycenone-treated mice (Fig. 4(a) and (l)). Next, ACC phosphorylation and FAS expression were measured, as they are downstream regulators of AMPK. ACC phosphorylation was significantly higher in the amycenone group (Fig. 4(b) and (m)), whereas FAS was significantly downregulated (Fig. 4(c) and (n)). Also, CaMKK, LKB1, Sirt1 and PKA, which are upstream factors of AMPK, were measured. While CaMKK did not change, LKB1 phosphorylation was significantly higher in amycenone-treated mice (Fig. 4(d), (e), (o) and (p)). Sirt1, an upstream regulator of LKB1, did not change between both control and treatment groups; however, PKA activation was significantly higher in the amycenone group (Fig. 4(f)–(h) and (q)–(s)). Finally, PPARγ, C/EBPα and C/EBPβ expression levels were unchanged by amycenone administration (Fig. 4(i)–(k) and (t)–(v)).

### Amycenone did not affect the expression levels of lipid metabolism-related genes in the liver of KK-*A^y^* mice

Furthermore, to investigate the effect of amycenone on the expression level of genes associated with lipid metabolism in the liver, the expression levels of the following genes were examined using real-time PCR: *Atgl*, *Aco*, *Mcad*, *Ppara*, *Cpt1*, *Adipor1*, *Adipor2*, *Tnf-a*, *Il-6* and *Il-1b*. Amycenone treatment did not affect all mRNA levels (Table 4).

**Table 4.** mRNA expression of lipid metabolism-related genes in the liver of KK-*A^y^* mice with normal water or under amycenone treatment (0.76 g/kg body weight/day) for 8 weeks

Genes	Control (%)	Amycenone (%)
*Atgl*	100⋅0 ± 0⋅22	111⋅6 ± 0⋅25
*Aco*	100⋅0 ± 0⋅30	111⋅7 ± 0⋅27
*Mcad*	100⋅0 ± 0⋅15	110⋅3 ± 0⋅24
*Ppara*	100⋅0 ± 0⋅13	92⋅6 ± 0⋅01
*Cpt1*	100⋅0 ± 0⋅16	72⋅3 ± 0⋅42
*Adipor1*	100⋅0 ± 0⋅04	86⋅5 ± 0⋅12
*Adipor2*	100⋅0 ± 0⋅16	79⋅6 ± 0⋅22
*Tnf-a*	100⋅0 ± 0⋅13	80⋅8 ± 0⋅51
*Il-6*	100⋅0 ± 0⋅13	93⋅6 ± 0⋅18
*Il-1b*	100⋅0 ± 0⋅27	83⋅3 ± 0⋅48

Amycenone did not affect gene expression in the liver of KK-*A^y^* mice. The table shows the gene expression levels of *Atgl*, *Aco*, *Mcad*, *Ppara*, *Cpt1*, *Adipor1*, *Adipor2*, *Tnf-a*, *Il-6* and *Il-1b*. The data value is given as means ± sem (*n* 9, 8, respectively).

## Discussion

Treatment with amycenone could ameliorate body weight gain in KK-*A^y^* mice. Furthermore, serum and hepatic TG and TC levels decreased in the amycenone group, indicating that this extract may have therapeutic benefits against hyperlipidaemia, NAFLD and obesity. Therefore, to elucidate the mechanisms of action of amycenone, an analysis of lipid metabolism-related proteins and genes expression in PAT, MAT and liver of KK-*A^y^* mice was performed.

AMPK is a crucial enzyme of lipid metabolism^(42)^ and is regulated by several factors. PKA, an upstream regulator of LKB1, activates AMPK either directly or through LKB1 phosphorylation.

In PAT, FAS, which is a downregulated factor of AMPK and associates with lipogenesis, was not affected in amycenone-treated KK-*A^y^* mice. ACC and HSL are downstream factors of AMPK. While ACC inhibits lipogenesis by being phosphorylated, HSL is involved in lipolysis. In the present study, amycenone-enhanced ACC and HSL phosphorylation levels in PAT. However, Sirt1, an upstream regulator of LKB1^(43)^, CaMKK and LKB1, which are further upstream regulators of the AMPK pathway^(44)^, did not affect amycenone-treated KK-*A^y^* mice of PAT. Thus, these findings revealed that amycenone can inhibit lipogenesis and lipolysis through PKA-AMPK-ACC and HSL pathways in PAT.

Furthermore, C/EBPβ phosphorylation, which is involved in lipolysis, also increased in the PAT of the amycenone-treated group. *Cpt1* is regulated via the AMPK-ACC pathway in lipid metabolism^(45–47)^. *Cpt1* is a rate-determining enzyme that promotes β-oxidation by transferring the long-chain fatty acid acyl-CoA to mitochondria^(48)^. It has been reported that the regulation mechanism of *Cpt1* expression via the AMPK-ACC pathway contributes to the promotion of lipolysis^(49)^. In the present study, the mRNA level of *Cpt1* increased in the amycenone-treated group. These results indicated that amycenone could promote mitochondrial β-oxidation. Therefore, amycenone promotes lipogenesis, lipolysis and mitochondrial β-oxidation through PKA-AMPK-ACC-*Cpt1* and HSL signal activation in PAT of KK-*A^y^* mice.

In MAT, amycenone induced PKA, AMPK and ACC phosphorylation. PPARγ is a transcription factor involved in lipogenesis and contributes to fat accumulation. In the present study, PPARγ expression level was significantly reduced in the amycenone-treated group. It can be concluded that amycenone inactivated lipogenesis through PKA-AMPK-ACC signalling and suppressing PPARγ expression level in MAT of KK-*A^y^* mice.

In summary, amycenone reduces excess body weight by promoting lipolysis and β-oxidation in both PAT and MAT through PKA-AMPK-ACC-*Cpt1* and HSL signal activation in the lipid metabolism pathway. Moreover, amycenone enhanced the C/EBPβ phosphorylation level in PAT and suppressed PPARγ expression in MAT. Thus, the differences observed between PAT and MAT mechanisms may depend on the type and part of the tissue.

Additionally, AMPK phosphorylation levels in EAT and SAT of KK-*A^y^* mice were investigated; however, AMPK phosphorylation did not significantly differ in EAT and SAT in mice under amycenone supplementation. Due to the different locations of each adipose tissue, there were no changes in AMPK phosphorylation between EAT and SAT. Therefore, PAT and MAT were selected for this study.

Next, because both hepatic TC and TG levels decreased after amycenone treatment in KK-*A^y^* mice, the effects of amycenone on lipid metabolism-related proteins and genes in the liver were analysed. The LKB1-AMPK signalling pathway was significantly activated, and PKA phosphorylation levels were high, indicating that amycenone enhanced lipid metabolism by inducing the PKA-LKB1-AMPK pathway. Moreover, exposure to amycenone phosphorylated ACC and downregulated FAS. These results indicated that amycenone could ameliorate hepatic fat accumulation by inhibiting lipogenesis through the activation of PKA-LKB1-AMPK-ACC and FAS signalling pathways in the liver.

Another possibility is that amycenone inhibits the absorption of nutrients in the intestinal tract. The results of the present study showed that administration of amycenone to KK-*A^y^* mice suppressed body weight gain and activated lipid metabolism pathways. Even if the absorption of nutrients is inhibited, there is almost no effect, and it is considered that the effect of amycenone is not lost.

This way, amycenone is shown to improve metabolic profiles, such as serum lipids, body weight gain and hepatic fat accumulation, by inhibiting lipogenesis and promoting lipolysis and β-oxidation in KK-*A^y^* mice. It is necessary to study amycenone as a novel component against obesity and hyperlipidaemia, although future clinical studies will be required to further confirm this clinical outcome.

## Conclusion

**Fig. 5.**
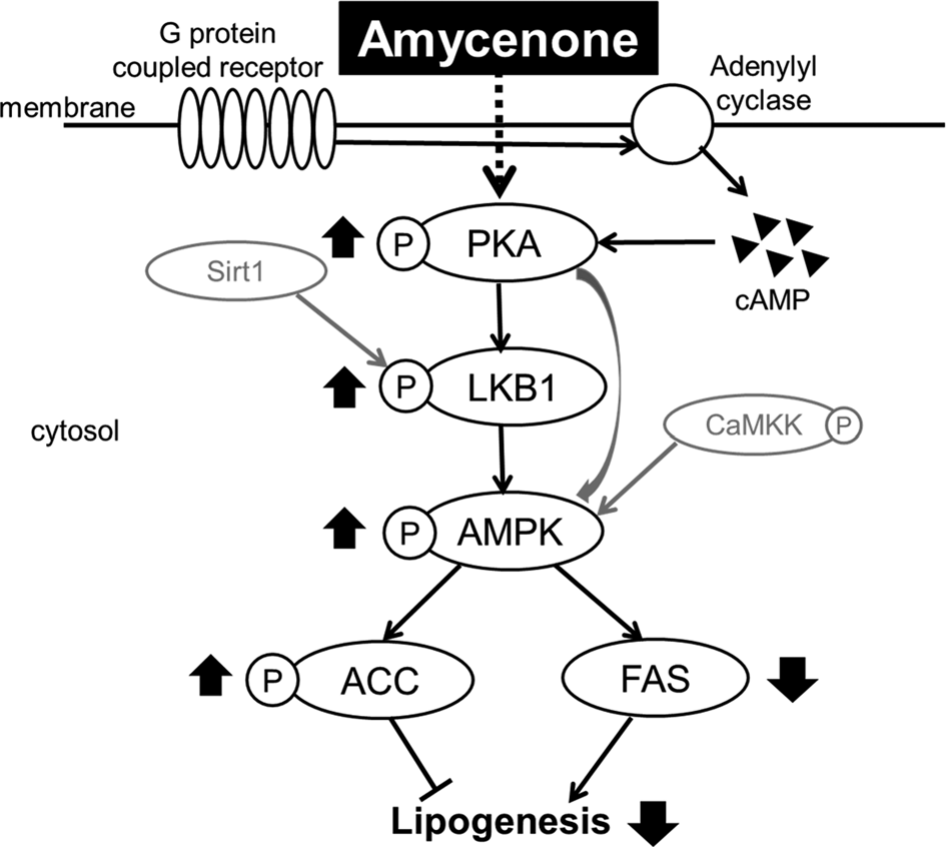
Signalling related to the reduction of hepatic fat accumulation in the liver of KK-*A^y^* mice with normal water or under amycenone treatment (0.76 g/kg body weight/day) for 8 weeks.

Conclusively, amycenone reduced body weight gain and serum and hepatic lipid levels in obese/diabetic KK-*A^y^* mice. The reduced body weights are due to amycenone-based inhibition of lipogenesis and fatty acid β-oxidation via PKA-AMPK-ACC-*Cpt1* and HSL pathways and reduction of lipogenesis via the PKA-AMPK-ACC pathway in PAT and MAT (Supplementary Fig. S4(a) and (b)). Besides, amycenone could improve hyperlipidaemia by reducing lipogenesis through PKA-LKB1-AMPK-ACC and FAS pathways in the liver (Fig. 5). Moreover, the phosphorylation level of C/EBPβ, a lipolysis-related transcription factor, was higher in PAT and the expression of PPARγ, a lipogenesis-related transcription factor, was lower in the MAT of amycenone-treated KK-*A^y^* mice. The results of the present study indicate that amycenone has an anti-obesity effect by suppressing body weight gain. The administration of amycenone to obese patients not only improves obesity but also treats and reduces the onset of lifestyle-related diseases, such as hypertension, hyperlipidaemia and diabetes, which are caused by excessive body weight gain. Furthermore, since amycenone is a natural product, it has fewer side effects caused by long-term administration of the drug and is expected to be developed as a safe treatment method.

## Supplementary material

For supplementary material accompanying this paper visit https://doi.org/10.1017/jns.2022.43. click here to view supplementary material
