# Supplementary material for: Amycenone reduces excess body weight and attenuates hyperlipidaemia by inhibiting lipogenesis and promoting lipolysis and fatty acid β-oxidation in KK-Ay obese diabetic mice
Source: J Nutr Sci. 2022 Jul 11;11:e55. doi: 10.1017/jns.2022.43 (PMC9274390; doi:10.1017/jns.2022.43)
Supplement: Supplementary file 1 [file jnssup.zip › S204867902200043Xsup001.zip!/The_latest_Supplementary_file(Journal_of_Nutritional Science).docx]

Research Article

**Amycenone reduces excess body weight and attenuates hyperlipidemia by inhibiting lipogenesis and promoting lipolysis and fatty acid β-oxidation in KK-*A^y^* obese diabetic mice**

**Shortened version of the title**

Amycenone reduces excess body weight in KK-*A^y^* mice

Maya Kudo,^#a^ Misa Hayashi,^a^ Boju Sun,^c,d^ Lili Wu,^e^ Tonghua Liu,^c,d^ Ming Gao*^a,b^

a *School of Pharmaceutical Science, Mukogawa Women’s University. 11-68 Koshien Kyuban-cho, Nishinomiya, Hyogo 663-8179, Japan*

b *Institute for Bioscience, Mukogawa Women’s University. 11-68 Koshien Kyuban-cho, Nishinomiya, Hyogo 663-8179, Japan*

c *Second Clinical Medical College, Beijing University of Chinese Medicine. No. 11 North Third Ring East Road, Beijing 100029, China*

d *Key Laboratory of Health Cultivation of the Ministry of Education, Beijing University of Chinese Medicine. No. 11 North Third Ring East Road, Beijing 100029, China*

e *College of traditional Chinese Medicine. Beijing University of Chinese Medicine. No. 11 North Third Ring East Road, Beijing 100029, China*

**Author contributions**

***Corresponding author: Ming Gao (MG), PhD**

Professor, Mukogawa Women’s University. 11-68 Koshien Kyuban-cho, Nishinomiya, Hyogo 663-8179, Japan

Email address: gaoming@mukogawa-u.ac.jp

Telephone and fax number: +81-798-45-9983

^#^**First author: Maya Kudo (MK)**

Email: 1422006@mwu.jp

**Authors’ email addresses**

MK: 1422006@mwu.jp

MH: hayasi27@mukogawa-u.ac.jp

BS: sunbucm@163.com

LW: gingnido_566@163.com

TL: thliu@vip.163.com

MG: gaoming@mukogawa-u.ac.jp

**Supplement file legends and table**

**Fig. S1 The phosphorylation levels of AMPK in EAT, SAT, PAT, and MAT of KK-*A^y^* mice with normal water or under amycenone treatment (0.76 g/kg body weight/day) for 8 weeks.**

Amycenone decreased the phosphorylation levels of AMPK in PAT and MAT of KK-*A^y^* mice. (A, G) EAT, (B, H) SAT, (C-E, I-K) PAT and (F, L) MAT. White bars represent the control group and black bars represent the amycenone group. The data value is given as means ± S.E.M. (n = 9, 8, respectively), **P* < 0.05, ***P* < 0.01 vs. control group.

**Fig. S2 The phosphorylation and expression levels of FAS, ACC, HSL, CaMKK, LKB1, PKA, and C/EBPβ in PAT of KK-*A^y^* mice with normal water or under amycenone treatment (0.76 g/kg body weight/day) for 8 weeks.**

Amycenone increased phosphorylation levels of ACC, HSL, and PKA in PAT of KK-*A^y^* mice. (A, H) FAS, (B, I) ACC, (C, J) HSL, (D, K) CaMKK, (E, L) LKB1, (F, M) PKA and (G, N) C/EBPβ. White bars represent the control group and black bars represent the amycenone group. The data value is given as means ± S.E.M. (n = 9, 8, respectively), **P* < 0.05, ***P* < 0.01 vs. control group.

**Fig. S3 The phosphorylation and expression levels of HSL, ACC, CaMKK, LKB1, Sirt1, PKA, PPARγ, C/EBPα, and C/EBPβ in MAT of KK-*A^y^* mice with normal water or under amycenone treatment (0.76 g/kg body weight/day) for 8 weeks.**

Amycenone enhanced phosphorylation levels of ACC, CaMKK, and PKA and reduced the expression level of PPARγ in MAT of KK-*A^y^* mice. (A, J) HSL, (B, K) ACC, (C, L) CaMKK, (D, M) LKB1, (E, N) Sirt1, (F, O) PKA, (G, P) PPARγ, (H, Q) C/EBPα and (I, R) C/EBPβ. White bars represent the control group and black bars represent the amycenone group. The data value is given as means ± S.E.M. (n = 9, 8, respectively), **P* < 0.05, ***P* < 0.01 vs. control group.

**Fig. S4 Signaling related to the inhibition of body weight gain in PAT and MAT of KK-*A^y^* mice with normal water or under amycenone treatment (0.76 g/kg body weight/day) for 8 weeks (A), (B).**

**Table S1 mRNA expression levels of lipid metabolism-related genes in PAT of KK-*A^y^* mice with normal water or under amycenone treatment (0.76 g/kg body weight/day) for 8 weeks.**

| Genes | Control (%) | Amycenone (%) |
| --- | --- | --- |
| *Atgl* | 100.0±1.41 | 93.2±1.58 |
| *Mcad* | 100.0±2.36 | 114.2±0.92 |
| *ap2* | 100.0±0.43 | 153.0±0.51 |
| *Cpt1* | 100.0±1.10 | 1196.3±0.29* |
| *Adipor1* | 100.0±1.66 | 109.2±0.55 |
| *Adipor2* | 100.0±0.31 | 144.1±0.79 |

Amycenone increased the gene expression level of *Cpt1* in PAT of KK-*A^y^* mice. The table shows the genes expression levels of *Atgl*, *Mcad*, *ap2*, *Cpt1*, *Adipor1*, and *Adipor2*. The data value is given as means ± S.E.M. (n = 9, 8, respectively), **P* < 0.05 vs. control group.
