# Supplementary material for: Amycenone reduces excess body weight and attenuates hyperlipidaemia by inhibiting lipogenesis and promoting lipolysis and fatty acid β-oxidation in KK-Ay obese diabetic mice
Source: J Nutr Sci. 2022 Jul 11;11:e55. doi: 10.1017/jns.2022.43 (PMC9274390; doi:10.1017/jns.2022.43)
Supplement: Supplementary file 1 [file jnssup.zip › S204867902200043Xsup001.zip!/The_latest_Supplementary_file(Journal_of_Nutritional_Science).pdf]

Research Article

Amycenone reduces excess body weight and attenuates hyperlipidemia by inhibiting lipogenesis and promoting lipolysis and fatty acid β-oxidation in KK-A^y obese diabetic mice

Shortened version of the title

Amycenone reduces excess body weight in KK-A^y mice

Maya Kudo,[#a] Misa Hayashi,[a] Boju Sun,[c,d] Lili Wu,[e] Tonghua Liu,[c,d] Ming Gao*[a,b]

a *School of Pharmaceutical Science, Mukogawa Women's University. 11-68 Koshien Kyuban-cho, Nishinomiya, Hyogo 663-8179, Japan*

b *Institute for Bioscience, Mukogawa Women's University. 11-68 Koshien Kyuban-cho, Nishinomiya, Hyogo 663-8179, Japan*

c *Second Clinical Medical College, Beijing University of Chinese Medicine. No. 11 North Third Ring East Road, Beijing 100029, China*

d *Key Laboratory of Health Cultivation of the Ministry of Education, Beijing University of Chinese Medicine. No. 11 North Third Ring East Road, Beijing 100029, China*

e *College of traditional Chinese Medicine. Beijing University of Chinese Medicine. No. 11 North Third Ring East Road, Beijing 100029, China*

Author contributions

***Corresponding author: Ming Gao (MG), PhD**

Professor, Mukogawa Women's University. 11-68 Koshien Kyuban-cho, Nishinomiya, Hyogo 663-8179, Japan

Email address: gaoming@mukogawa-u.ac.jp

Telephone and fax number: +81-798-45-9983

[#]**First author: Maya Kudo (MK)**

Email: 1422006@mwu.jp

Authors' email addresses

MK: 1422006@mwu.jp

MH: hayasi27@mukogawa-u.ac.jp

BS: sunbucm@163.com

LW: gingnido_566@163.com

TL: thliu@vip.163.com

MG: gaoming@mukogawa-u.ac.jp

Supplement file legends and table

Fig. S1 The phosphorylation levels of AMPK in EAT, SAT, PAT, and MAT of KK-*A^y^* mice with normal water or under amycenone treatment (0.76 g/kg body weight/day) for 8 weeks.

Amycenone decreased the phosphorylation levels of AMPK in PAT and MAT of KK-A^y mice. (A, G) EAT, (B, H) SAT, (C-E, I-K) PAT and (F, L) MAT. White bars represent the control group and black bars represent the amycenone group. The data value is given as means ± S.E.M. (n = 9, 8, respectively), *$P < 0.05$, **$P < 0.01$ vs. control group.

Fig. S2 The phosphorylation and expression levels of FAS, ACC, HSL, CaMKK, LKB1, PKA, and C/EBPβ in PAT of KK-A^y mice with normal water or under amycenone treatment (0.76 g/kg body weight/day) for 8 weeks.

Amycenone increased phosphorylation levels of ACC, HSL, and PKA in PAT of KK-A^y mice. (A, H) FAS, (B, I) ACC, (C, J) HSL, (D, K) CaMKK, (E, L) LKB1, (F, M) PKA and (G, N) C/EBPβ. White bars represent the control group and black bars represent the amycenone group. The data value is given as means ± S.E.M. (n = 9, 8, respectively), *$P < 0.05$, **$P < 0.01$ vs. control group.

Fig. S3 The phosphorylation and expression levels of HSL, ACC, CaMKK, LKB1, Sirt1, PKA, PPARγ, C/EBPα, and C/EBPβ in MAT of KK-*A*^{y} mice with normal water or under amycenone treatment (0.76 g/kg body weight/day) for 8 weeks.

Amycenone enhanced phosphorylation levels of ACC, CaMKK, and PKA and reduced the expression level of PPARγ in MAT of KK-*A*^{y} mice. (A, J) HSL, (B, K) ACC, (C, L) CaMKK, (D, M) LKB1, (E, N) Sirt1, (F, O) PKA, (G, P) PPARγ, (H, Q) C/EBPα and (I, R) C/EBPβ. White bars represent the control group and black bars represent the amycenone group. The data value is given as means ± S.E.M. (n = 9, 8, respectively), $*P < 0.05$, $**P < 0.01$ vs. control group.

Fig. S4 Signaling related to the inhibition of body weight gain in PAT and MAT of KK-*A*^{y} mice with normal water or under amycenone treatment (0.76 g/kg body weight/day) for 8 weeks (A), (B).

Table S1 mRNA expression levels of lipid metabolism-related genes in PAT of KK-*A*^{y} mice with normal water or under amycenone treatment (0.76 g/kg body weight/day) for 8 weeks.

Genes	Control (%)	Amycenone (%)
Atgl	100.0±1.41	93.2±1.58
Mcad	100.0±2.36	114.2±0.92
ap2	100.0±0.43	153.0±0.51
Cpt1	100.0±1.10	1196.3±0.29*
Adipor1	100.0±1.66	109.2±0.55
Adipor2	100.0±0.31	144.1±0.79

Amycenone increased the gene expression level of *Cpt1* in PAT of KK-*A*^{y} mice. The table shows the genes expression levels of *Atgl*, *Mcad*, *ap2*, *Cpt1*, *Adipor1*, and *Adipor2*. The data value is given as means ± S.E.M. (n = 9, 8, respectively), $*P < 0.05$ vs. control group.
